# Revealing tumor cells and tissues with high selectivity through folic acid-targeted nanofluorescence probes responsive to acidic microenvironments

**DOI:** 10.3389/fonc.2024.1404148

**Published:** 2024-06-12

**Authors:** Jing Li, Hongyi He, Shuyan Liu, Xining Li, Fengfeng Wu

**Affiliations:** ^1^ Neurobiology Laboratory, Wannan Medical College, Wuhu, China; ^2^ College of Pharmacy, Hubei University of Science and Technology, Xianning, China; ^3^ Department of Obstetrics and Gynecology, Second Hospital of Jilin University, Changchun, China; ^4^ School of Medicine, Huzhou University, Huzhou, China; ^5^ Department of Orthopedics and Rehabilitation, Huzhou Hospital of Zhejiang University School of Medicine, Huzhou, China; ^6^ Department of Orthopedics and Rehabilitation, Huzhou Central Hospital, Huzhou, China

**Keywords:** tumor cells, tissues, folic acid, nanofluorescence, probes, acidic microenvironments

## Abstract

Tumor-specific fluorescent probes must fulfill the dual requirements of targeted accumulation within tumors and high-resolution imaging capabilities. To achieve both tumor-targeted accumulation and high-resolution imaging performance, we developed a composite comprising an acid-responsive bodipy conjugated to amphiphilic PEG-b-PLA polymer, along with folic acid (FA)-modified PEG-b-PLA as a targeting moiety for active tumor-specific accumulation. Finally, a novel assembly of hybrid fluorescent nanoparticles was successfully synthesized by integrating these two components, demonstrating exceptional responsiveness to acidic conditions for fluorescence excitation and remarkable tumor-targeted accumulation capabilities. We conducted comprehensive *in vitro* and *in vivo* investigations employing techniques such as analysis of physicochemical properties, fluorescence-based probes detection at varying pH levels, assessment of *in vitro* cytotoxicity, evaluation of cellular uptake capacity, analysis of lysosomal co-localization imaging, examination of tumor fluorescence images *in vivo*, and investigation of biological distribution patterns. The results demonstrated that the acid-responsive nanofluorescence probe we designed and synthesized possesses desirable physical and chemical characteristics, including a small particle size and low cytotoxicity. Moreover, it exhibits rapid real-time response to acidic environments and displays enhanced fluorescence intensity, enabling the real-time tracking of probe entry into tumor cells as well as intracellular lysozyme accumulation. We achieved highly specific *in vivo* tumor visualization by combining nanoprobes targeting folate receptor. Through imaging cervical tumor mice, we demonstrated the precise imaging performance and high targeted accumulation of FA-targeted nanofluorescence probes in tumor tissue. Furthermore, we confirmed the *in vivo* safety of the FA-targeted nanofluorescence probe through biological distribution analysis. These findings highlight the potential widespread application of FA-targeted acid-responsive nanofluorescence probes for selective imaging of tumor cells and tissues.

## 1 Introduction

Fluorescence imaging has emerged as a convenient and safe tracer technique for labeling various tissues in organisms (1–3). To date, numerous fluorescent probes have been developed, with 4,4-difluoro-4-bora-3a,4a-diaza-s-indacene (BDY) garnering significant attention owing to its robust ultraviolet absorption, exceptional physical stability, high fluorescence peak intensity, adjustable emission properties, and superior quantum yield (4). Despite the extensive development of BDY-based probes for diverse biomedical applications (5), their tumor targeting distribution and specific imaging efficiency often fall short of ideal expectations. The reason can be attributed to the challenges encountered by BDY, a small molecule fluorescent probe, which are similar to those faced by other small molecule fluorescent probes. For instance, the majority of small molecule fluorescent probes exhibit hydrophobic properties and are readily absorbed by the reticuloendothelial system following intravenous administration (6), consequently leading to a reduced half-life in blood circulation. Furthermore, the utilization of small molecule fluorescent probes often necessitates their conjugation with various biological entities such as antibodies, peptides, and nucleic acids to achieve a certain level of targeting specificity (7–9). However, such binding interactions significantly compromise the sensitivity of *in vivo* probe detection. Consequently, there is an urgent imperative to develop highly tumor-sensitive fluorescent probes possessing superior tumor-targeting properties.

Currently, nanoparticles are widely employed for the efficient transportation of fluorescent probes in various applications (10). In comparison to conventional biomarker techniques, nanoprobes offer several advantages including uniform particle size distribution, stable optical properties, minimal cytotoxicity, excellent water solubility, and remarkable biocompatibility (11). Therefore, they exhibit enhanced resolution, heightened fluorescence intensity, and superior detection capabilities. Specifically, PLA-b-PEG nanocarriers possess the advantages of excellent biocompatibility, biodegradability, and prolonged *in vivo* circulation time, rendering them extensively employed for the transportation of fluorescent probes. Generally, nanoparticles can passively target tumor sites through their enhanced permeability and retention effect by delivering various probes (12, 13). Additionally, many types of nanoparticles are surface-modified with specific targeting groups to enhance their accumulation in tumors while reducing uptake in normal tissues (14–17). The same approach can also be employed for the delivery of fluorescent probes, thereby endowing fluorescent nanoparticles with not only enhanced tumor tissue targeting capabilities but also heightened sensitivity in detecting tumor tissues. The achievement of this goal necessitates addressing two pivotal issues: firstly, the fluorescence emission of nanoparticles in normal tissues should be suppressed until their internalization by tumor tissue and subsequent exposure to acidic environments within the tumor, thereby enabling significant specific fluorescence emission. Another crucial aspect that necessitates attention is the effective integration of tumor-specific highly expressed substances as targeting moieties with nanoprobes, thereby augmenting their accumulation within tumors while minimizing uptake in normal tissue. To address these dual challenges concurrently, this study proposes the utilization of two carriers to fabricate multifunctional composite nanoparticles. One carrier is required to load a substantial quantity of acid-sensitive components, while the other carrier needs to accommodate a significant number of targeted groups.

To achieve this objective, we synthesized two polymers by combining an acid-responsive dye (BDY) or folate receptor-targeting ligand (FA) with amphiphilic polyethylene glycol-b-polylactic acid (PEG-b-PLA) polymer. Subsequently, these components were assembled into composite fluorescent nanoparticles, and their physical and chemical properties, pH-dependent fluorescence emission behavior, *in vitro* cytotoxicity spectrum, cellular uptake efficiency in tumor cells, lysosomal co-localization imaging ability, as well as comprehensive analysis of *in vitro* and *in vivo* tumor fluorescence images were investigated.

## 2 Materials and methods

### 2.1 Materials

The following reagents were obtained from Sigma-Aldrich Co. (St. Louis, MO, USA): N-hydroxysuccinimide (NHS), N,N-diisopropylethylamine (DIPEA), 2,3-dichloro-5,6-dicyano-1,4-benzoquinone (DDQ), N,N’-dicyclohexylcarbodiimide (DCC), dimethylformamide (DMF), tetrahydrofuran (THF). Additionally, FA and N-acetyl-L-cysteine were also purchased from Sigma-Aldrich Co. Always-on probes were procured from GlpBio (Cat. No.: GC66041, USA).

### 2.2 Synthesis of pH-activatable nanoprobes

Dissolve 2,4-dimethylpyrrole and the corresponding 4-substituted benzaldehyde in dichloromethane, followed by conducting a condensation reaction. After stirring the resulting reddish solution overnight, DDQ was introduced and the mixture was stirred for 2 h. Subsequently, DIPEA and BF_3_OEt_2_ were added to the reaction mixture. After an additional 1 h of reaction time, the mixture was concentrated under reduced pressure and then redissolved in EtOAc before being washed with water. The aqueous layer was extracted with EtOAc, while the combined organic layers were dried, filtered, and subsequently concentrated under reduced pressure. Finally, purification of the residue using silica gel column chromatography yielded BDY1.

The synthesis of BDY2 was achieved through the Vilsmeier-Haack reaction. In brief, a mixture of DMF and POCl_3_ was stirred at 0°C for 5 min, followed by warming to room temperature and stirring for an additional 30 min. Subsequently, BDY2 in dichloroethane was added to the mixture and stirred at 50°C for 2 h. After cooling to room temperature, the mixture was washed with NaHCO_3_ and water. The organic layers were then evaporated and dried under vacuum conditions. Finally, purification of the crude product was accomplished using column chromatography resulting in the isolation of pure BDY2.

The synthesis of BDY2-PEG-b-PLA was conducted as follows: NH_2_-PEG-b-PLA and BDY2 were dissolved in THF and stirred at room temperature overnight. Subsequently, NaBH_4_ was added and the mixture was stirred for an additional 12 h. The resulting product was purified through dialysis in methanol for 24 h followed by freeze-drying for subsequent use.

### 2.3 Preparation of FA-targeted pH-activatable nanoprobes

At 0°C, DCC and NHS were added to DMF dissolved with FA and stirred for 12 h. Subsequently, NH_2_-PEG-b-PLA was added and the mixture was stirred for an additional 12 h. The resulting solution was concentrated, precipitated in ether, collected, and dried under vacuum. Hybrid micelles were also prepared using co-assembly methods. Briefly, BDY2-PEG-b-PLA and FA-PEG-b-PLA were dissolved in THF followed by sonication for 2 min. The polymer solutions were then dropwise added to deionized water while vigorously stirring the suspension for 10 h until no THF residue could be detected.

### 2.4 *In vitro* release

The nanoprobes were suspended in phosphate buffer saline (PBS) with a pH of 7.4, containing Tween-80 at a concentration of 1.0 wt%. Subsequently, the solution was added to a dialysis bag with a molecular weight cutoff of 3.5 kDa for release testing purposes. Briefly, the sealed dialysis bag was placed inside a vial and immersed in PBS. The vial was then subjected to shaking on a table at a frequency of 1 Hz and maintained at 37°C. At regular intervals of every 2 h, medium samples were withdrawn from the vial and replaced with an equal volume of fresh buffer solution. The amount of BDY released was determined through UV-vis analysis using a detection wavelength set at 500 nm.

### 2.5 Fluorescence detection of probes

The fluorescence intensity of always-on probes and pHAN was measured in acidic (pH=4.4), neutral (pH=7.4), and basic (pH=8.4) PBS solutions, respectively. The excitation wavelength used was 488 nm, and emission spectra were obtained from 515 nm onwards. Unmixed images were generated by employing authentic spectral patterns while considering the background.

### 2.6 Cell line and cell culture

The human cervical epithelial cell line (HCerEpic cells) and human cervical carcinoma cell line (HeLa cells) were obtained from the cell bank of the Shanghai Institutes for Biological Sciences. The cells were cultured at 37°C in a humidified atmosphere with 5% (v/v) CO_2_ using Dulbecco’s Modified Eagle Medium (DMEM) supplemented with 10% fetal bovine serum, as well as streptomycin (100 U/mL) and penicillin (100 mg/mL).

### 2.7 Cytotoxicity test

Cells were seeded in 96-well plates at a density of 1×10^6^ cells/well and incubated in DMEM for 24 h. Subsequently, the medium was replaced with various concentrations of probes and further incubated for 48 h. Following this, 3-(4,5-Dimethylthiazol-2-yl)-2,5- diphenyltetrazolium bromide (MTT) solution (5 mg/mL) in PBS was added to each well and the plates were incubated for an additional 4 h at 37°C. The culture medium containing MTT was then removed, and dimethyl sulfoxide was added to dissolve the formazan crystals in each well. Finally, the plates were shaken for 10 min and the absorbance of formazan product was measured at 570 nm using a microplate reader (Bio-RAD Model 680).

### 2.8 Confocal laser scanning microscopy

Sterile coverslips were placed in a six-well plate at a density of 2×10^4^ cells/well, and HeLa cells were cultured at 37°C for 24 h until they adhered to the well. The probes were then co-cultivated with HeLa cells for 0–8 h at 37°C. After washing the cells three times with PBS (pH=7.4), probe uptake was observed using CLSM (FluoView FV1000, Olympus). To investigate the co-localization phenomenon between the probe and lysosome, the prepared LysoTracker solution was added to the treated cells and incubated at 37°C for 30 min. Subsequently, the cells were fixed with 4% paraformaldehyde at 4°C for 10 min and washed twice with PBS. The nuclei were stained by adding a solution of 4’,6-diamidino-2-phenylindole (DAPI) in PBS, followed by gentle washing to remove excess dyes. Finally, CLSM was employed to capture images of each culture dish after co-culturing the cells and probes for 4 h. In order to evaluate the tumor cell targeting function of FA on the surface of FA-pHAN, we pre-incubated the cells with free FA (4 mM) for 30 min prior to introducing FA-pHAN. Subsequently, we added FA-pHAN to block the binding between the probes’ FA and its receptors on tumor cells, thereby impeding the targeted effect of FA-pHAN on tumor cells. CLSM was employed to capture images of each culture dish after co-culturing the cells and probes for 4 h.

### 2.9 *In vivo* fluorescence imaging

Animal experiments were conducted in accordance with the National Institutes of Health standards and the Guidelines for the Care and Use of Experimental Animals, and were approved by the Animal Experiment Ethics Committee of Huzhou University. Cervical tumors were induced in Kunming mice through subcutaneous injection of 2×10^6^ HeLa cells suspended in 200 μL of PBS. Comparative experiments were then conducted using nude mice bearing HeLa cervical tumors to directly compare always-on probes, pHAN, and FA-pHAN for specificity monitoring at a post-injection time point of 2 h. For tumor imaging, pHAN (10 mg/kg) or FA-pHAN (10 mg/kg) was intravenously administered to the tumor-bearing mice. To compare probe accumulation in normal and tumor tissues, we injected the same probe into two different sites of each mouse-namely, the tumor tissue on one leg and the corresponding normal tissue on the opposite leg. Subsequently, euthanasia anesthesia was used to sacrifice the mice. The excitation wavelength for probe activation was set at 488 nm, and emission spectra were obtained at 515 nm. Unmixed images were generated using actual spectral patterns and backgrounds. By comparing average fluorescence intensity between the tumor site and whole body excluding the tumor site, we determined the ratio of tumor to normal tissue (T=N).

### 2.10 Biological distribution

After 24 h of administration, the major organs and tumors were dissected, weighed, and their measurements recorded. Each tissue sample was placed in an EP tube and homogenized with physiological saline using a homogenizer. Subsequently, methanol and chloroform were added separately and vortexed for 5 min. The lower liquid phase was collected after centrifugation. Following natural evaporation, dimethyl sulfoxide was added for re-dissolution. The upper clear liquid phase was then quantified based on the established fluorescence standard curve to calculate the Bodipy content in each sample.

### 2.11 Statistical analysis

All data are presented as the mean ± standard deviation and were analyzed using one-way analysis of variance followed by Tukey’s *post-hoc* test. A *P*-value of less than 0.05 was considered to indicate a statistically significant difference.

## 3 Results

### 3.1 Physical and chemical properties of probes

**Scheme 1:**
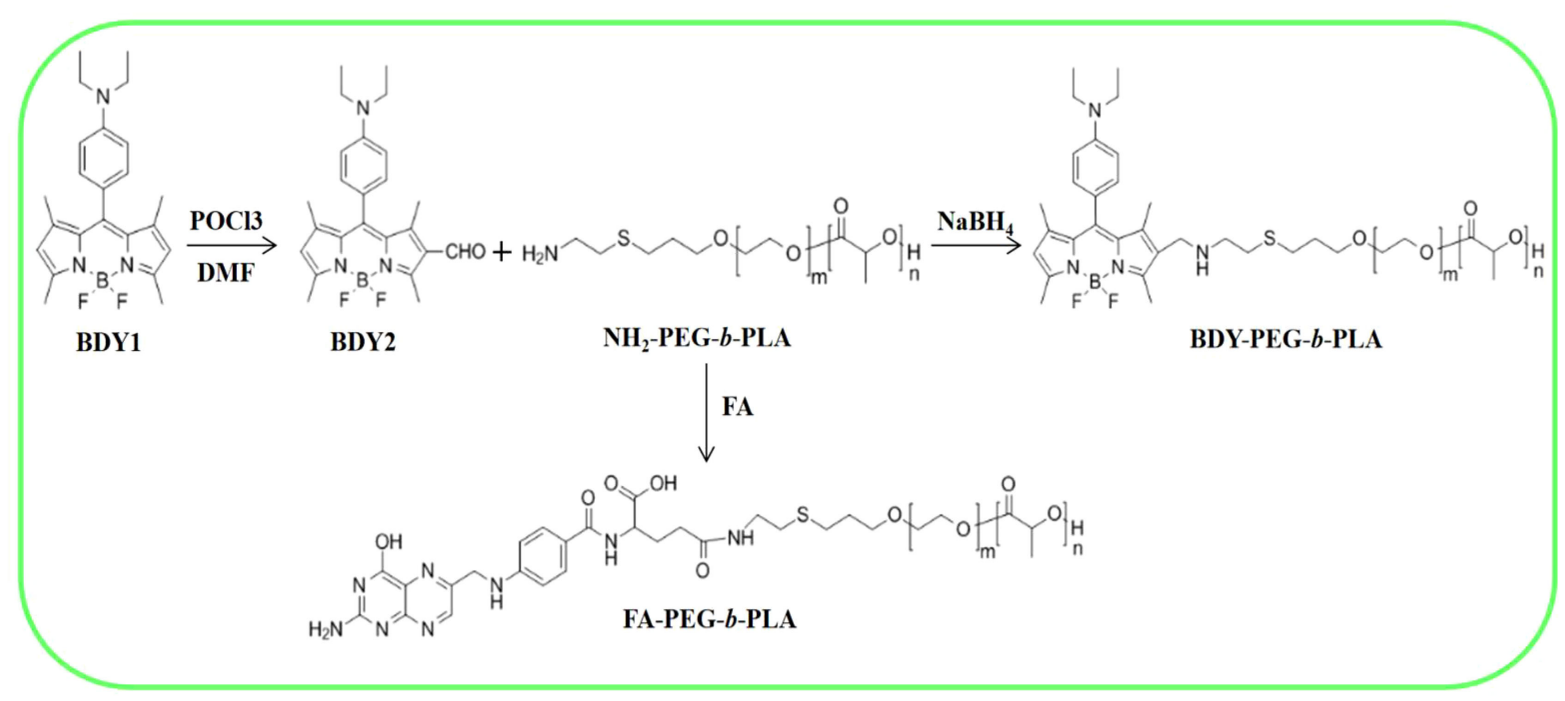
Design strategy for nanoprobes with pH-responsiveness and tumor targeting.

**Scheme 2:**
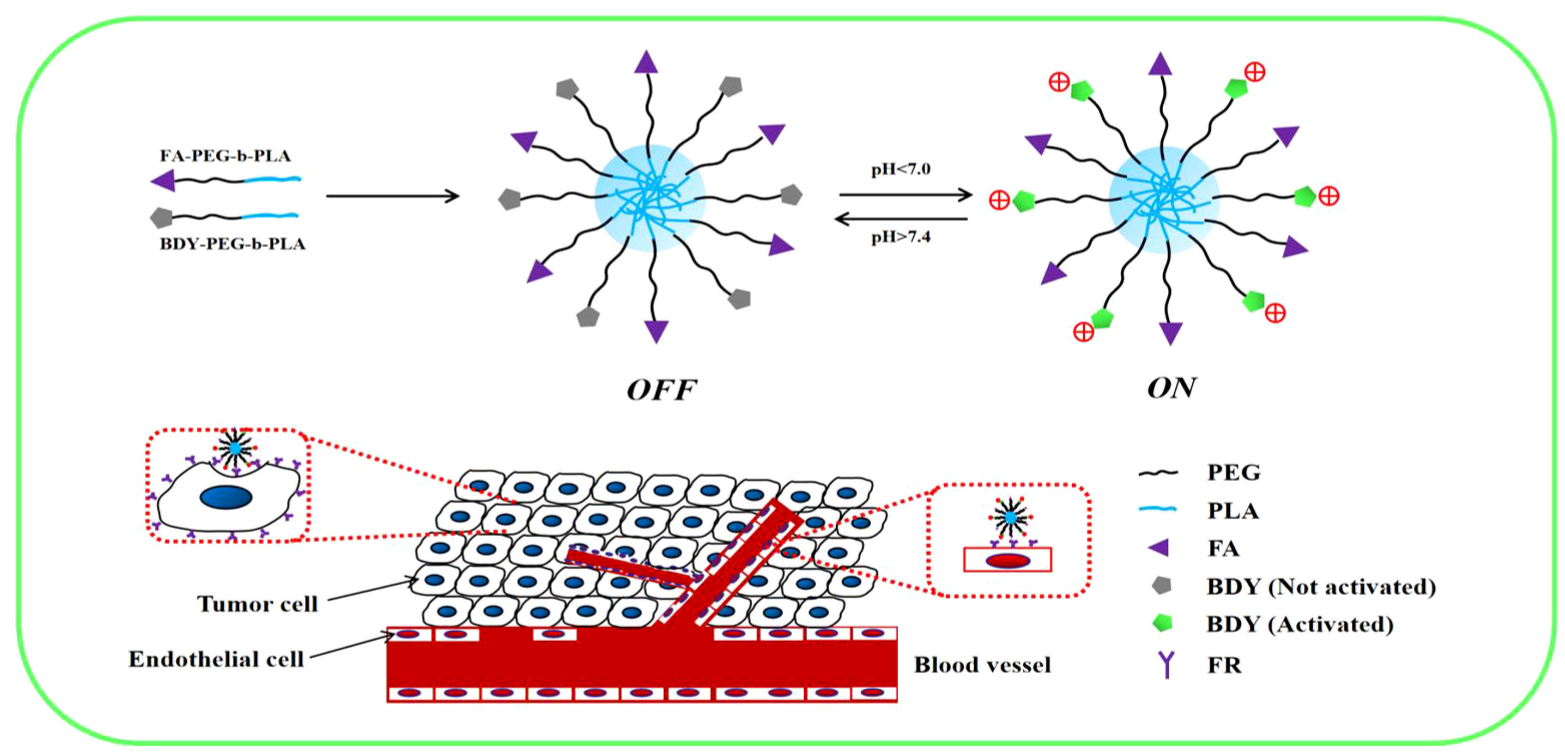
Synthesis process of BDY2-PEG-b-PLA and FA-PEG-b-PLA.

**Figure 1:**
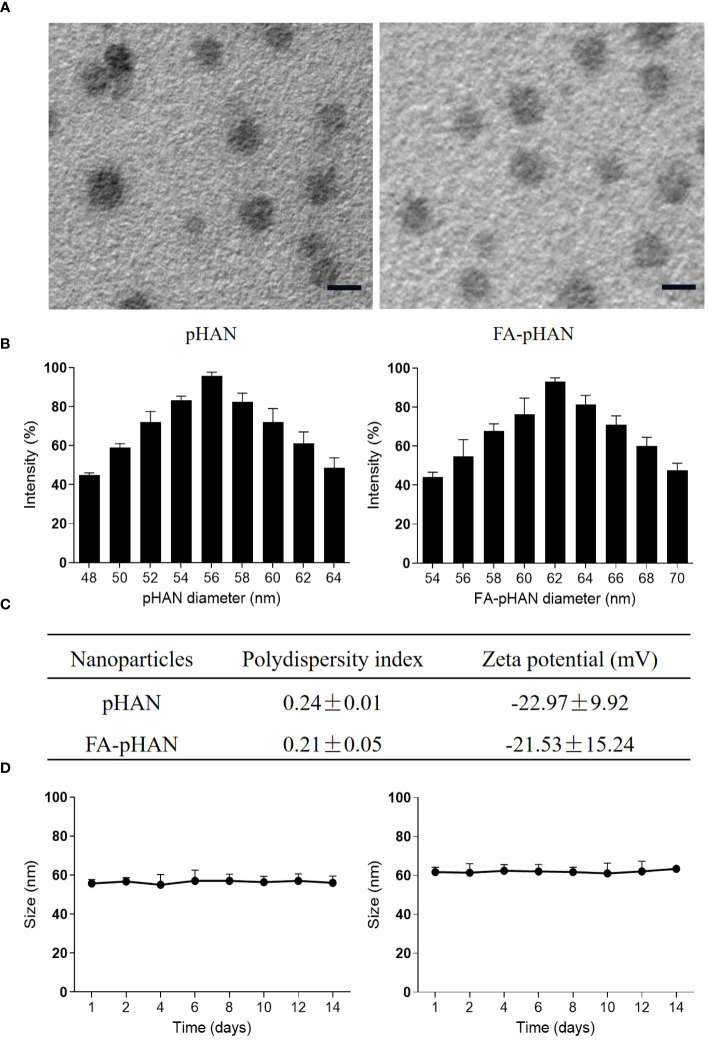
**(A)** TEM images of pHAN and FA-pHAN, with a scale bar of 50 nm. **(B)** Diameter of pHAN and FA-pHAN. **(C)** Polydispersity index and zeta potential measurements of the nanoparticles. **(D)** The size of pHAN and FA-pHAN undergoes changes over time. pHAN, pH-activatable nanoprobes; FA-pHAN, FA-targeted pH-activatable nanoprobes.

**Figure 2:**
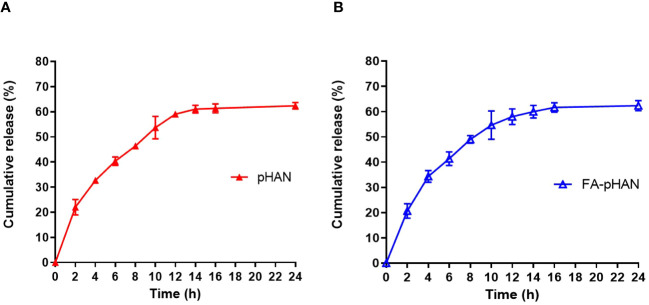
Cumulative release profile of the fluorescent dye BDY from pHAN **(A)** and FA-pHAN **(B)**. pHAN, pH-activatable nanoprobes; FA-pHAN, FA-targeted pH-activatable nanoprobes.

**Figure 3:**
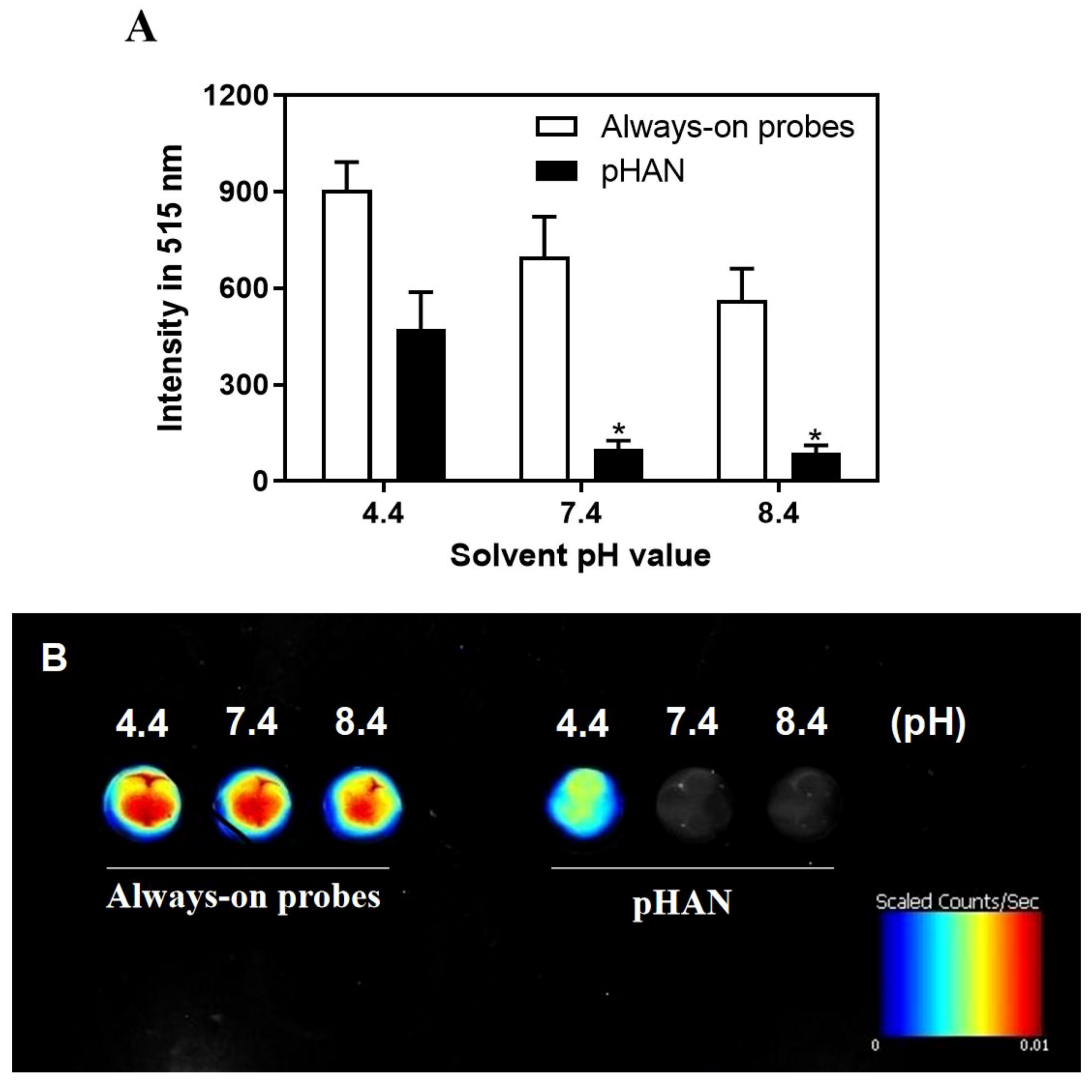
**(A)** Fluorescence intensity comparison between always-on probes and pHAN at different pH values, showing changes in fluorescence spectra under acidic conditions (pH=4.4), neutral conditions (pH=7.4), and basic conditions (pH=8.4). * *P* < 0.05 compared to the pH 4.4 group for statistical significance analysis purposes only. **(B)** Fluorescence imaging comparison between always-on probes and pHAN. pHAN, pH-activatable nanoprobes; FA-pHAN, FA-targeted pH-activatable nanoprobes.

As depicted in **Scheme 1**, we synthesized two copolymers, namely BDY2-PEG-b-PLA and FA-PEG-b-PLA, for the assembly of FA-pHAN. BDY2 was utilized to react with the amino group of NH_2_-PEG-b-PLA, forming a schiff base which could be subsequently reduced to stable carbon nitrogen bonds by NaBH_4_ treatment, resulting in the formation of BDY2-PEG-b-PLA. Additionally, employing the DCC/NHS method, we conjugated the target molecule FA onto NH_2_-PEG-b-PLA polymer to generate FA-PEG-b-PLA. As illustrated in **Scheme 2**, an acid-responsive component comprising BDY coupled with amphiphilic PEG-b-PAL polymer was prepared and further modified with FA as an active targeting moiety to yield another component possessing active targeting capability. Finally, fluorescent nanoparticles were assembled from these two components and exhibited both acid-responsive fluorescence excitation ability and tumor-targeted accumulation performance. As depicted in **Figures 1A**, **B**, both pHAN and FA-pHAN exhibit typical spherical morphology with diameters of 56 nm and 62 nm, respectively, while demonstrating exceptional monodispersity in aqueous solutions. The specific physical and chemical parameters of the two copolymers and nanoparticles are presented in **Figure 1C**. The BDY2 compound, which contains aldehyde groups, was synthesized via the Vilsmeier-Haack reaction with a yield of 80%. Composite nanoparticles were prepared by mixing BDY2-PEG-b-PLA with FA-PEG-b-PLA to achieve pH-responsive properties and tumor tissue targeting. The mixed nanoparticles had a FA content of 20wt%. These results further demonstrate that the nanoprobes we developed meet the requirements of an ideal drug delivery system. The diameters of pHAN and FA-pHAN, as measured by DLS, remained relatively constant over a period of 2 weeks (see **Figure 1D**). The release of pHAN and FA-pHAN is depicted in **Figure 2**, illustrating a gradual and sustained release of BDY from both materials. Within 14 h, approximately 60% of BDY was released cumulatively. Subsequently, the release rate for both probes decelerated, eventually reaching a plateau after 16 h. To validate the pH-dependent fluorescence emission characteristics of the probe, we conducted measurements of fluorescence intensity across various pH values. The probe exhibited maximum absorption at approximately 515 nm. As depicted in **Figures 3A**, **B**, the fluorescence intensity of the always-on probes demonstrated no discernible correlation with the pH levels present within the buffer. The fluorescence intensity of pHAN, in contrast, exhibits a decrease as the pH increases, indicating an absence of fluorescence at physiological pH and a progressively increasing trend at lower pH levels.

### 3.2 Cytotoxicity of probes

**Figure 4:**
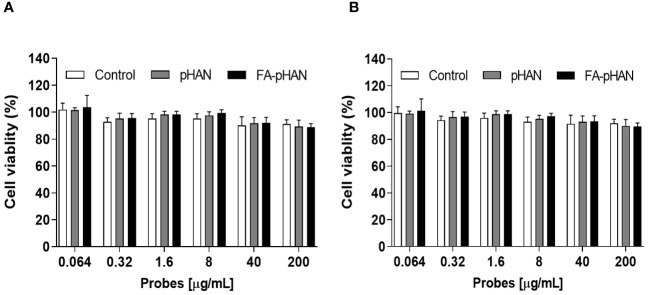
*In vitro* cytotoxicity of pHAN and FA-pHAN against HCerEpic cells **(A)** and HeLa cells **(B)** at 48 h. pHAN, pH-activatable nanoprobes; FA-pHAN, FA-targeted pH-activatable nanoprobes.

The MTT method was employed to investigate the potential cytotoxicity of probes on HCerEpic cells and HeLa cells at doses ranging from 0.064 to 200 μg/mL, as depicted in **Figure 4**. Following a co-culture with pHAN and FA-pHAN for 48 h, the viability of HCerEpic cells and HeLa cells remained above 90% irrespective of whether pHAN or FA-pHAN was administered at low or high doses, thereby indicating negligible *in vitro* cytotoxicity associated with pHAN and FA-pHAN during cell experiments.

### 3.3 Cell uptake of probes

**Figure 5:**
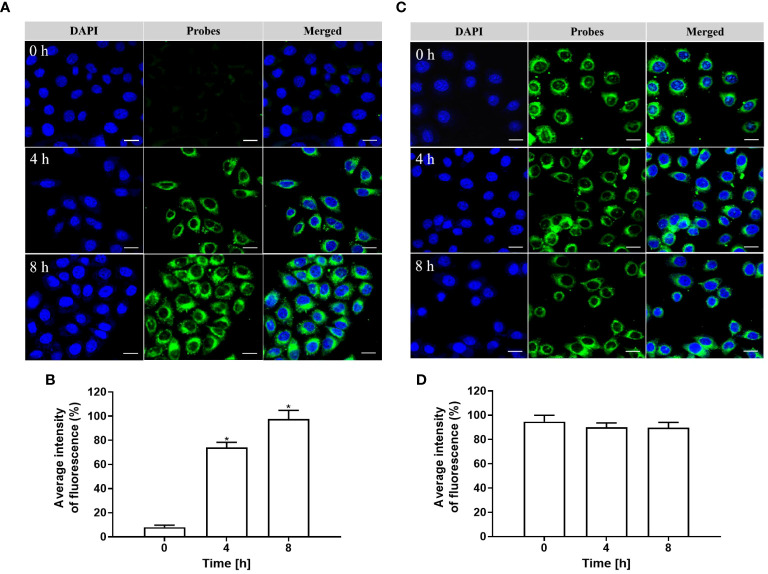
**(A)** CLSM images of HeLa cells cultured at 37°C with pHAN for 0 h, 4 h, and 8 h are shown. The green channel represents pHAN fluorescence, while the blue channel shows DAPI staining. A scale bar of 20 μm is included for reference. **(B)** Quantitative analysis was performed on HeLa cells cultured at 37°C with pHAN for a duration of 0–8 h (*P < 0.05 compared to the 0 h group). **(C)** CLSM images depict HeLa cells cultured at 37°C with always-on probes for time points of 0 h, 4 h, and 8 h. The green channel corresponds to always-on probes fluorescence, while the blue channel indicates DAPI staining. A scale bar of 20 μm is provided as a size reference. **(D)** Quantitative analysis was conducted on HeLa cells cultured at a temperature of 37°C using always-on probes over a period ranging from 0–8 h.V pHAN, pH-activatable nanoprobes.

As depicted in **Figures 5A**, **B**, the cells were co-cultured with pHAN for a duration of 0–8 h. As the phagocytosis of pHAN by the cells progressed, there was a significant enhancement in fluorescence signals at 4 h of incubation compared to 0 h, and stronger fluorescence signals were observed at 8 h of incubation compared to 4 h. Conversely, as illustrated in **Figures 5C**, **D**, nearly identical green fluorescence was detected in the cells after co-culturing with the always-on probes for a duration of 0–8 h. These findings suggest that pHAN exhibits substantial intracellular accumulation and fluorescence activation starting from approximately 4 h post-ingestion by tumor cells.

**Figure 6:**
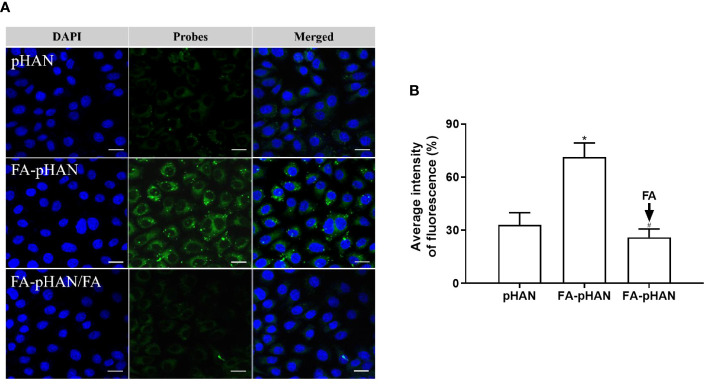
**(A)** CLSM images of HeLa cells incubated with pHAN, FA-pHAN, or competitive assay with free FA added 10 min prior to FA-pHAN administration. Green channel represents pHAN or FA-pHAN, while blue channel shows DAPI staining. Scale bar: 20 μm. **(B)** Quantitative analysis of HeLa cells treated with pHAN, FA-pHAN, or FA-pHAN/FA for 4 h. *P < 0.05 compared to the pHAN group; # *P* < 0.05 compared to the FA-pHAN group. pHANm, pH-activatable nanoprobes; FA-pHAN, FA-targeted pH-activatable nanoprobes.

To elucidate the efficacy of targeting functional group FA in facilitating cellular uptake of probes, we incubated cells with pHAN or FA-pHAN for 4 h and assessed intracellular fluorescence signals. As depicted in **Figures 6A**, **B**, the fluorescence intensity observed in the FA-pHAN group was significantly higher compared to that of the pHAN group, indicating that FA labeling can expedite cell internalization of pHAN. Pre-incubation of free FA in the cell culture medium resulted in reduced uptake of FA-pHAN by cells, exhibiting a fluorescence intensity similar to that observed in the pHAN uptake group. This data further substantiates that enhanced cellular uptake of FA-pHAN is attributed to specific interactions between FA and its receptors on tumor cells.

### 3.4 Lysosome colocalization imaging of probes

**Figure 7:**
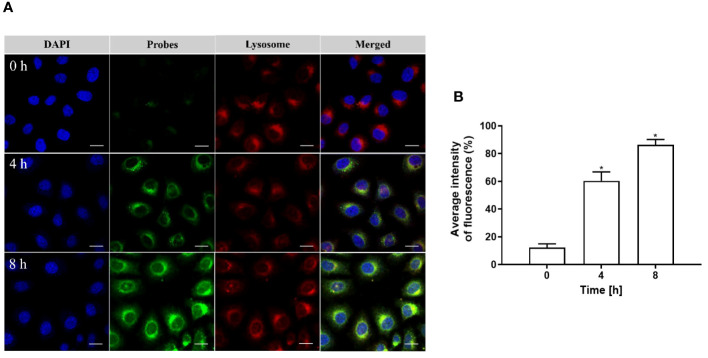
**(A)** CLSM images depicting the co-localization of pHAN with lysosomes in HeLa cells cultured at 37°C for 0 h, 4 h, and 8 h. The green channel represents pHAN, the red channel represents lysosomes, and the blue channel represents DAPI-stained nuclei. Scale bar: 20 μm. **(B)** Quantitative analysis demonstrating the degree of co-localization between pHAN and lysosomes. *P < 0.05 compared to the 0 h group. pHAN, pH-activatable nanoprobes.

Upon engulfment by cells, pHAN gradually accumulates within lysosomes, where its green fluorescence is activated due to the acidic nature of these organelles. The resulting yellow fluorescence arises from the superimposition of the green fluorescence emitted by pHAN probes and the red fluorescence emitted by lysosomes. As depicted in **Figures 7A**, **B**, negligible green fluorescence was observed in cells after co-culturing with pHAN for 0 h. However, as the co-culture time extended to 4 h and 8 h, enhanced green and yellow fluorescence became evident in HeLa cells. Subsequent colocalization studies revealed that these yellow fluorescent spots were predominantly localized within acidic lysosomes.

### 3.5 Fluorescence images of tumor bearing mice

**Figure 8:**
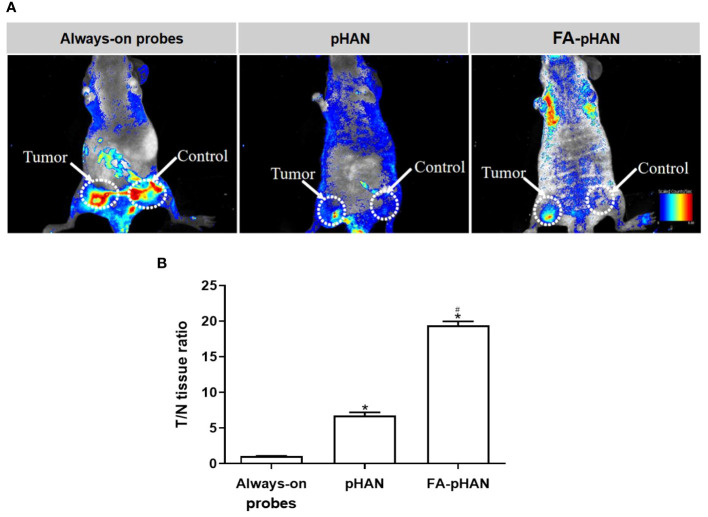
**(A)** Overlaid fluorescent images of HeLa-tumor-bearing mice at 2 h post-injection of always-on probes, pHAN and FA-pHAN. **(B)** Near-infrared fluorescence intensity ratio between tumor and normal tissues (T=N ratio) as a function of probes injection. *P < 0.05 compared to the pHAN group; # *P* < 0.05 compared to the FA-pHAN group. pHAN, pH-activatable nanoprobes; FA-pHAN, FA-targeted pH-activatable nanoprobes.

Fluorescence imaging was conducted 2 h post intravenous administration of each probe group in mice with subcutaneously transplanted HeLa cells. As depicted in **Figure 8**, a significant fluorescence signal was observed exclusively at the tumor site in mice injected with pHAN, while no fluorescence emission was detected in normal tissues. Conversely, the always-on probes group exhibited substantial fluorescence signals both in tumor and normal tissues. The fluorescence intensity observed within the tumor tissue of FA-pHAN injection group mice increased by a factor of 11 compared to the pHAN group, whereas no notable fluorescence signal was discernible in normal tissue.

### 3.6 Biological distribution of probes in major organs

**Figure 9:**
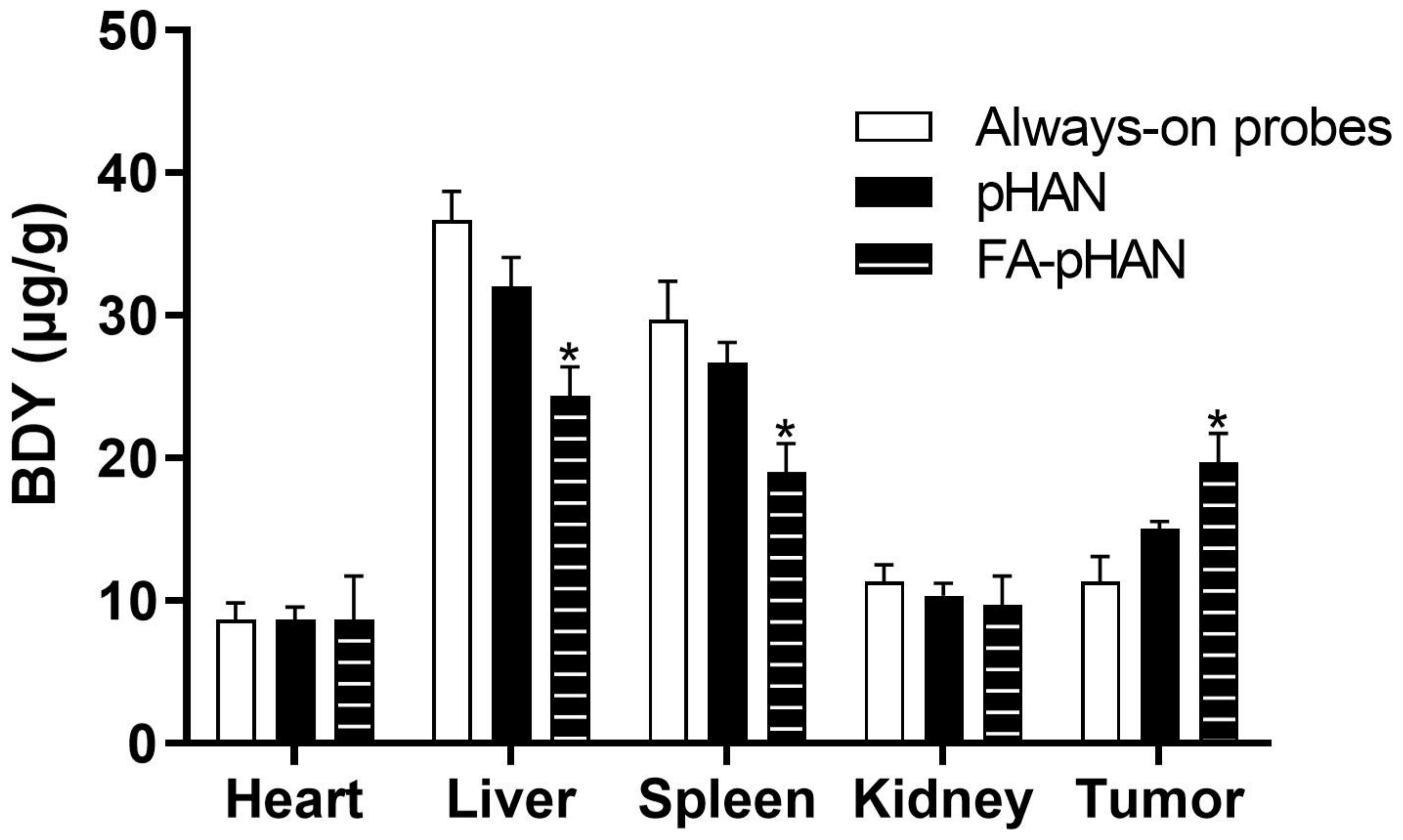
The biodistribution of mice at 24 h post-injection of always-on probes, pHAN and FA-pHAN. *P < 0.05 compared to the always-on probes group. pHAN, pH-activatable nanoprobes; FA-pHAN, FA-targeted pH-activatable nanoprobes.

To investigate the *in vivo* activity of the probe, we conducted an examination of the organ distribution of the drug in animal subjects. In this study, mice were administered the drug for a period of 24 h. The findings, depicted in **Figure 9**, reveal a higher presence of permanently open probes in the liver and spleen. When compared to always-on probes, there was a slight reduction in the distribution of pHAN within these organs, while FA-pHAN exhibited a significant decrease. Notably, there was a slight increase in pHAN content within tumor tissue and a substantial increase in FA-pHAN content within tumor tissue when compared to that observed with the always-on probe. This observation suggests that targeted accumulation of nanoprobes featuring specific targeting groups at tumor sites may serve to diminish probe distribution within other organs while concurrently enhancing their accumulation within tumor tissues.

## 4 Discussion

Recently, multifunctional nanoparticles have garnered significant attention in various applications such as biosensors, nanoprobes, and targeted drug delivery. These endeavors primarily stem from the imperative need for precise control of drug release to enhance biological specificity in diagnosis and treatment. To accomplish this objective, researchers have been dedicated to developing stimulus-responsive nano platforms that can be triggered by environmental cues including pH variations, enzyme expression levels, redox reactions, and light sources (18–20). Among these activation signals, the pH difference between tumor tissue and normal tissue or various organelles within tumor cells has garnered widespread attention as a stimulation mode for inducing fluorescence activation of nanoprobes (21). Several pH-responsive nanosystems have been reported to enhance the sensitivity or therapeutic effectiveness of tumor imaging (21–24). In addition, folate receptors are overexpressed on the surface of many malignant tumor cells (25, 26), while they are rarely or only slightly expressed in normal cells (27). Therefore, by modifying the vector’s surface with FA as the targeting group based on the characteristics of folic acid receptor expression, targeted delivery to tumor cells with overexpressed folic acid receptors can be achieved. This approach enables successful drug delivery to tumor cells, thus minimizing toxicity to normal cells and enhancing drug efficacy (28). However, further research is required to achieve targeted transport of nanoprobes to tumor cells and their specific activation within these cells. Therefore, our objective is to develop nanoprobes that possess both pH-responsiveness and tumor cell targeting capabilities.

The BDY exhibits strong visible light absorption, high fluorescence quantum yield, and excellent photostability (4). In this study, we employed BDY with aniline in the middle position as the substrate for pHAN. To verify the pH-dependent fluorescence characteristics of the probe, we measured the fluorescence intensity emitted by both pHAN and always-on probes at various pH values. Interestingly, only pHAN showed significant changes in fluorescence intensity upon altering the pH. Conversely, the fluorescence intensity of pHAN is significantly influenced by pH, with no observable fluorescence at physiological pH; however, a notable increase in fluorescence occurs at lower pH levels. These findings suggest that pHAN can serve as a responsive imaging tool in acidic tumor environments. Additionally, we assessed the potential cytotoxicity of nanoprobes on cells using the MTT method and determined that any potential toxic effects from both pHAN and FA-pHAN were negligible *in vitro* cell experiments. To investigate the intracellular uptake ability of always-on probes and pHAN, CLSM was employed to detect the cellular uptake of probes in HeLa cells. It was observed that upon co-culturing with pHAN for 0 h, minimal green fluorescence was detected within the cells compared to the control sample. However, as the co-culture time extended to 4 h and particularly 8 h, a significant increase in green fluorescence intensity was observed in HeLa cells. Conversely, when co-cultured with always-on probes for 0–8 h, consistent levels of green fluorescence were detected within the cells. These findings suggest that pHAN may gradually accumulate within specific acidic organelles subsequent to internalization by tumor cells, resulting in its fluorescence being progressively stimulated by the acidic microenvironment. This hypothesis will be validated in forthcoming investigations. Lysosomes are intracellular compartments housing acid hydrolases responsible for waste degradation and cellular debris breakdown (29). Therefore, it is imperative to conduct further investigation into the lysosomal localization of pHAN. To facilitate observation, nuclear staining of HeLa cells was performed using DAPI, which emits blue fluorescence. Additionally, specific labeling of lysosomes was achieved using LysoTracker ^®^ Red DND 99, emitting red fluorescence. Our findings demonstrate a gradual accumulation of pHAN within lysosomes upon cellular engulfment and its activation in response to acidic environments, resulting in an increasing emission of green fluorescence. Subsequently, the green fluorescence emitted by the probe and the red fluorescence originating from the lysosome overlap, resulting in a distinct yellow fluorescence signal. As anticipated, these prominent yellow spots predominantly localized within acidic lysosomal compartments at both 4 h and 8 h time points. This data strongly suggests that a significant proportion of pHAN molecules are internalized by tumor cells and subsequently sequestered within acidic lysosomes, thereby facilitating efficient emission of high-intensity fluorescence in response to acidity and ultimately achieving pH-responsive fluorescent behavior.

In the design of multifunctional nanoprobes, while the responsiveness to the tumor acidic environment is crucial, it is equally important to ensure their efficient accumulation at the tumor site. Insufficient accumulation may hinder effective discrimination between tumor and normal tissue, thereby compromising both sensitive imaging and targeted functionality. Hence, simultaneous achievement of tumor tissue-sensitive imaging and targeting functions remains a pivotal challenge that necessitates immediate attention. To accomplish this objective, we synthesized FA-pHAN based on the pHAN architecture. In the subsequent experimental investigation, CLSM was employed to observe the fluorescence activation level of FA-pHAN within tumor cells. The findings from this study demonstrate that following a 4 h incubation period with nanoprobes, the fluorescence signal emitted by FA-pHAN is significantly higher compared to non-targeted pHAN. However, early addition of free FA in the cell culture medium diminishes the cellular uptake of FA-pHAN, indicating that enhanced internalization of FA-pHAN by cells is attributed to the interaction between FA and tumor cells. The results suggest that FA-pHAN exhibits excellent tumor cell targeting ability and holds great potential as a promising candidate for tumor-targeted imaging. Our *in vivo* tumor imaging studies have further validated the precise delivery of FA-pHAN to the target site, with its fluorescence being effectively activated under acidic conditions within the tumor microenvironment, while no such changes were observed in normal tissues, thus enabling successful differentiation between healthy and diseased tissues. Furthermore, our biodistribution analysis demonstrated that FA-pHAN exhibits a targeted accumulation in tumor tissue compared to always-on probes or pHAN. This also leads to a reduction in probe accumulation in the liver and spleen, thereby minimizing potential side effects associated with *in vivo* administration and improving the targeted anti-cancer efficacy.

In summary, we have successfully engineered and synthesized pHAN with exceptional pH responsiveness, enabling significant activation within acidic organelles. This nanoplatform offers an efficient ON/OFF system for tumor imaging and drug delivery. Furthermore, to achieve tumor targeting functionality, we further developed FA-pHAN based on this platform. *In vitro* and *in vivo* studies unequivocally demonstrated that the fluorescence signal delivery efficiency of FA-pHAN surpassed that of non-targeted pHAN in both tumor cells and tissues. Our findings underscore the remarkable tumor cell targeting ability and specific imaging capability of FA-pHAN, positioning it as a promising candidate for real-time monitoring of tumor cells and tissues.

## Data availability statement

The raw data supporting the conclusions of this article will be made available by the authors, without undue reservation.

## Ethics statement

The animal study was approved by The Ethics of Animal Research Ethics Committee of Huzhou University. The study was conducted in accordance with the local legislation and institutional requirements.

## Author contributions

JL: Methodology, Investigation, Formal analysis, Writing – original draft. HH: Methodology, Investigation, Formal analysis, Writing – original draft. SL: Methodology, Investigation, Formal analysis, Writing – original draft. XL: Investigation, Formal analysis, Conceptualization, Methodology, Resources, Writing – review & editing. FW: Conceptualization, Methodology, Formal analysis, Resources, Writing – original draft, Writing – review & editing, Supervision.

## References

[1] Hesemans E Buttiens K Manshian BB Soenen SJ. The role of optical imaging in translational nanomedicine. J Funct Biomater. (2022) 13:137. doi: 10.3390/jfb13030137 36135572 PMC9502568

[2] Cui FZ Liu JH Pang SW Li B. Recent advance in tumor microenvironment-based stimuli-responsive nanoscale drug delivery and imaging platform. Front Pharmacol. (2022) 13:929854. doi: 10.3389/fphar.2022.929854 35935835 PMC9354407

[3] Refaat A Yap ML Pietersz G Walsh APG Zeller J Del Rosal B. *In vivo* fluorescence imaging: success in preclinical imaging paves the way for clinical applications. J Nanobiotechnol. (2022) 20:450. doi: 10.1186/s12951-022-01648-7 PMC9571426 36243718

[4] Rybczynski P Smolarkiewicz-Wyczachowski A Piskorz J Bocian S Ziegler-Borowska M Kędziera D. Photochemical properties and stability of BODIPY dyes. Int J Mol Sci. (2021) 22:6735. doi: 10.3390/ijms22136735 34201648 PMC8267640

[5] Antina E Bumagina N Marfin Y Guseva G Nikitina L Sbytov D. BODIPY conjugates as functional compounds for medical diagnostics and treatment. Molecules. (2022) 27:1396. doi: 10.3390/molecules27041396 35209191 PMC8877204

[6] Zan CF An J Wu ZF Li SJ. Engineering molecular nanoprobes to target early atherosclerosis: Precise diagnostic tools and promising therapeutic carriers. Nanotheranostics. (2023) 7:327–44. doi: 10.7150/ntno.82654 PMC10093416 37064609

[7] Turner MA Lwin TM Amirfakhri S Nishino H Hoffman RM Yazaki PJ. The use of fluorescent anti-CEA antibodies to label, resect and treat cancers: A review. Biomolecules. (2021) 11:1819. doi: 10.3390/biom11121819 34944463 PMC8699160

[8] Xie RX Wu ZJ Zeng FX Cai HW Wang D Gu L. Retro-enantio isomer of angiopep-2 assists nanoprobes across the blood-brain barrier for targeted magnetic resonance/fluorescence imaging of glioblastoma. Signal Transduct Target Ther. (2021) 6:309. doi: 10.1038/s41392-021-00724-y 34413288 PMC8377144

[9] Vilímová L Hervé-Aubert K Chourpa L. Formation of miRNA nanoprobes-conjugation approaches leading to the functionalization. Molecules. (2022) 27:8428. doi: 10.3390/molecules27238428 36500520 PMC9739806

[10] Li W Kaminski Schierle GS Lei BF Liu YL Kaminski CF. Fluorescent nanoparticles for super-resolution imaging. Chem Rev. (2022) 122:12495–543. doi: 10.1021/acs.chemrev.2c00050 PMC9373000 35759536

[11] Prieto-Montero R Katsumiti A Cajaraville MP López-Arbeloa I Martínez-Martínez V. Functionalized fluorescent silica nanoparticles for bioimaging of cancer cells. Sensors (Basel). (2020) 20:5590. doi: 10.3390/s20195590 33003513 PMC7582890

[12] Tian HL Zhang TT Qin SY Huang Z Zhou L Shi JY. Enhancing the therapeutic efficacy of nanoparticles for cancer treatment using versatile targeted strategies. J Hematol Oncol. (2022) 15:132. doi: 10.1186/s13045-022-01320-5 36096856 PMC9469622

[13] Sharifi M Cho WC Ansariesfahani A Tarharoudi R Malekisarvar H Sari S. An updated review on EPR-based solid tumor targeting nanocarriers for cancer treatment. Cancers (Basel). (2022) 14:2868. doi: 10.3390/cancers14122868 35740534 PMC9220781

[14] Mahmoud K Swidan S El-Nabarawi M Teaima M. Lipid based nanoparticles as a novel treatment modality for hepatocellular carcinoma: a comprehensive review on targeting and recent advances. J Nanobiotechnol. (2022) 20:109. doi: 10.1186/s12951-022-01309-9 PMC8898455 35248080

[15] Yao HJ Sun L Li JC Zhou XF Li R Shao RG. A novel therapeutic siRNA nanoparticle designed for dual-targeting CD44 and Gli1 of gastric cancer stem cells. Int J Nanomed. (2020) 15:7013–34. doi: 10.2147/IJN.S260163 PMC7522319 33061365

[16] Li WP Little N Park JH Foster CA Chen JW Lu JQ. Tumor associated fibroblasts-targeting nanoparticles for enhancing solid tumor therapy: progresses and challenges. Mol Pharm. (2021) 18:2889–905. doi: 10.1021/acs.molpharmaceut.1c00455 PMC8752044 34260250

[17] Beh CY Prajnamitra RP Chen LL Hsieh PCH. Advances in biomimetic nanoparticles for targeted cancer therapy and diagnosis. Molecules. (2021) 26:5052. doi: 10.3390/molecules26165052 34443638 PMC8401254

[18] Rahim MA Jan N Khan S Shah H Madni A Khan A. Recent advancements in stimuli responsive drug delivery platforms for active and passive cancer targeting. Cancers (Basel). (2021) 13:670. doi: 10.3390/cancers13040670 33562376 PMC7914759

[19] Jin MZ Jin WL. The updated landscape of tumor microenvironment and drug repurposing. Signal Transduct Target Ther. (2020) 5:166. doi: 10.1038/s41392-020-00280-x 32843638 PMC7447642

[20] Firouzabadi BM Gigliobianco MR Joseph JM Censi R Martino PD. Design of nanoparticles in cancer therapy based on tumor microenvironment properties. Pharmaceutics. (2022) 14:2708. doi: 10.3390/pharmaceutics14122708 36559202 PMC9785496

[21] Siriwibool S Kaekratoke N Chansaenpak K Siwawannapong K Panajapo P Sagarik K. Near-infrared fluorescent pH responsive probe for targeted photodynamic cancer therapy. Sci Rep. (2020) 10:1283. doi: 10.1038/s41598-020-58239-5 31992821 PMC6987190

[22] Farjadian F Ghasemi S Akbarian M Hoseini-Ghahfarokhi M Moghoofei M Doroudian M. Physically stimulus-responsive nanoparticles for therapy and diagnosis. Front Chem. (2022) 10:952675. doi: 10.3389/fchem.2022.952675 36186605 PMC9515617

[23] Qiu KQ Du Y Liu JY Guan JL Chao H Diao JJ. Super-resolution observation of lysosomal dynamics with fluorescent gold nanoparticles. Theranostics. (2020) 10:6072–81. doi: 10.7150/thno.42134 PMC7254985 32483439

[24] Wang LJ Zhou Q Yang HY. A facile fabrication of lysosome-targeting pH fluorescent nanosensor based on PEGylated polyester block copolymer. Polymers (Basel). (2022) 14:2420. doi: 10.3390/polym14122420 35745996 PMC9231249

[25] Martín-Sabroso C Torres-Suárez AI Alonso-González M Fernández-Carballido A Fraguas-Sánchez AI. Active targeted nanoformulations via folate receptors: State of the Art and future perspectives. Pharmaceutics. (2022) 14:14. doi: 10.3390/pharmaceutics14010014 PMC8781617 35056911

[26] Jurczyk M Jelonek K Musiał-Kulik M Beberok A Wrześniok D Kasperczyk J. Single- versus dual-targeted nanoparticles with folic acid and biotin for anticancer drug delivery. Pharmaceutics. (2021) 13:326. doi: 10.3390/pharmaceutics13030326 33802531 PMC8001342

[27] Hu YW Chen DY Napoleon JV Srinivasarao M Singhal S Savran CA. Efficient capture of circulating tumor cells with low molecular weight folate receptor-specific ligands. Sci Rep. (2022) 12:8555. doi: 10.1038/s41598-022-12118-3 35595733 PMC9122947

[28] Mahalunkar S Yadav AS Gorain M Pawar V Braathen R Weiss S. Functional design of pH-responsive folate-targeted polymer-coated gold nanoparticles for drug delivery and *in vivo* therapy in breast cancer. Int J Nanomedicine. (2019) 14:8285–302. doi: 10.2147/IJN.S215142 PMC6801194 31802866

[29] Webb BA Aloisio FM Charafeddine RA Cook J Wittmann T Barber DL. pHLARE: A new biosensor reveals decreased lysosome pH in cancer cells. Mol Biol Cell. (2021) 32:131–42. doi: 10.1091/mbc.E20-06-0383 PMC8120692 33237838

